# Structure–Mechanical Relationships in Alginate–Chitosan Polymer Composites

**DOI:** 10.3390/polym18060713

**Published:** 2026-03-15

**Authors:** Hatice Sıçramaz

**Affiliations:** Department of Food Engineering, Faculty of Engineering, Sakarya University, Sakarya 54050, Turkey; haticesicramaz@sakarya.edu.tr

**Keywords:** alginate–chitosan composites, polyelectrolyte complexation, crosslinking, structure–mechanical relationships, hydrogel networks, mechanical properties

## Abstract

Alginate–chitosan composites are widely used bio-based materials due to their biocompatibility, biodegradability, and relatively simple processing methods. By combining the complementary properties of alginate and chitosan, these systems offer adjustable mechanical characteristics suitable for applications such as tissue engineering, wound healing, drug delivery, and sustainable packaging. However, although many studies report improved mechanical properties, the link between structural design and mechanical behavior is often discussed within specific applications rather than examined in a broader context. This review focuses on how polymer ratio, charge balance, crosslinking strategy, reinforcement approach, and processing conditions influence the mechanical properties of alginate–chitosan composites. Instead of considering these factors separately, the available studies are discussed in terms of how the internal structure of the composite affects stiffness, strength, deformability, and stability. This review brings together findings from various fields to highlight shared structure–mechanical relationships and to provide guidance for designing alginate–chitosan composites with specific mechanical properties.

## 1. Introduction

Natural polysaccharide-based materials have attracted growing interest as sustainable alternatives to synthetic polymers. Their popularity stems mainly from their biocompatibility, biodegradability, and the possibility of being processed under relatively mild and environmentally friendly conditions [1,2]. Among these materials, alginate and chitosan stand out because they are widely available, show low toxicity, and readily form hydrogel structures [3,4].

Alginate, an anionic polysaccharide derived from brown seaweed, forms ionically crosslinked gels in the presence of divalent cations such as Ca^2+^ [5]. Chitosan, obtained by partial deacetylation of chitin, carries positively charged amino groups that promote intermolecular interactions and pH-responsive behavior [6,7]. Despite these advantages, using alginate or chitosan alone often results in materials with limited mechanical strength, excessive swelling, or insufficient long-term stability [4,8]. For this reason, combining both polymers into composite systems have become an attractive strategy to overcome these limitations.

Combining alginate and chitosan into composite systems provides a more robust and stable polymer structure through the formation of polyelectrolyte complexes driven by electrostatic interactions between oppositely charged functional groups [9]. As a result, alginate–chitosan composites have been investigated in a variety of fields. Applications include tissue engineering [3,10,11], wound healing systems [12,13,14], controlled drug delivery [15,16,17], and environmentally friendly packaging [18,19]. Although many studies report enhanced mechanical properties, the results differ widely depending on polymer ratio, molecular characteristics, crosslinking method, and processing conditions [4,20,21]. Because findings are often presented within specific application contexts, it remains difficult to identify broader design principles that link structure to mechanical performance.

Several reviews have addressed alginate- or chitosan-based materials individually or have focused primarily on specific applications of alginate–chitosan systems [2]. In contrast, a systematic, structure-oriented evaluation of how network architecture governs mechanical behavior across different composite designs remains limited. Addressing this gap is essential for translating alginate–chitosan composites from laboratory-scale formulations into reliable, application-oriented materials.

Accordingly, this review examines structure–mechanical property relationships in alginate–chitosan composites. By bringing together experimental studies from different application areas, it discusses how polymer composition, intermolecular interactions, crosslinking strategies, reinforcement approaches, and processing conditions influence mechanical performance. For this purpose, literature published mainly between 2010 and 2025 was examined using databases including Web of Science, Scopus, PubMed, and ScienceDirect. The search was conducted using combinations of keywords such as “alginate–chitosan composite”, “polyelectrolyte complex”, “hydrogel mechanical properties”, “Young’s modulus”, “tensile strength”, “compressive modulus”, “swelling ratio”, “crosslinking”, “polymer ratio”, “network structure”, “FTIR”, and “zeta potential”. The initial search yielded a large number of publications, which were subsequently screened for relevance based on their focus on structure–mechanical relationships and experimentally reported mechanical parameters. After removing duplicates and studies outside the scope, the most relevant articles were selected to support the analysis presented in this review, while earlier foundational studies were included where necessary to explain key interaction mechanisms. This review differs from previous reviews by focusing specifically on structure–mechanical property relationships and design strategies in alginate–chitosan composites rather than providing only application-oriented summaries.

## 2. Mechanical Behavior of Alginate–Chitosan Composites

Mechanical performance of alginate–chitosan composites is typically evaluated using compression, tensile, and rheological tests, which provide complementary information on stiffness, strength, and viscoelastic behavior of the polymer network. The mechanical behavior of alginate–chitosan composites depends not only on the intrinsic properties of the individual polymers but also on how they interact and how the material is processed. Because alginate and chitosan carry opposite charges and exhibit different gelation mechanisms, their combination makes it possible to create composite structures with properties that neither polymer can achieve alone. Alginate typically provides rapid ionic gelation and an initial structural framework, whereas chitosan contributes additional cohesion and environmental responsiveness [4]. Understanding how composition, crosslinking mechanisms, and external conditions affect the resulting structure is essential for designing composites with targeted mechanical properties.

### 2.1. Influence of Polymer Composition

**Figure 1:**
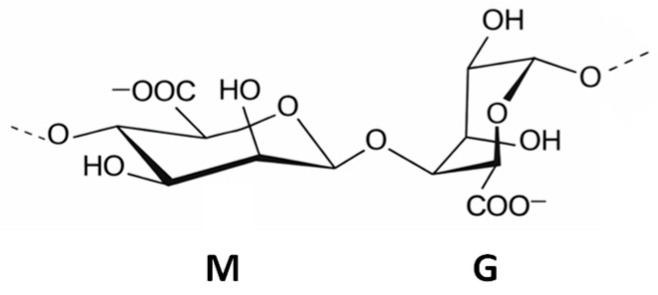
Chemical structure of alginate composed of mannuronic (M) and guluronic (G) units.

Alginate is a linear anionic polysaccharide composed of β-D-mannuronic (M) and α-L-guluronic (G) acid residues arranged in different block sequences (Figure 1). The relative content and distribution of these blocks strongly influence gel formation and mechanical behavior. G-rich alginates generally form stiffer but more brittle gels because of stronger and more cooperative ionic crosslinking with divalent cations, often described by the “egg-box” model. In contrast, M-rich alginates tend to produce softer and more flexible structures due to increased chain mobility [4,22].

**Figure 2:**
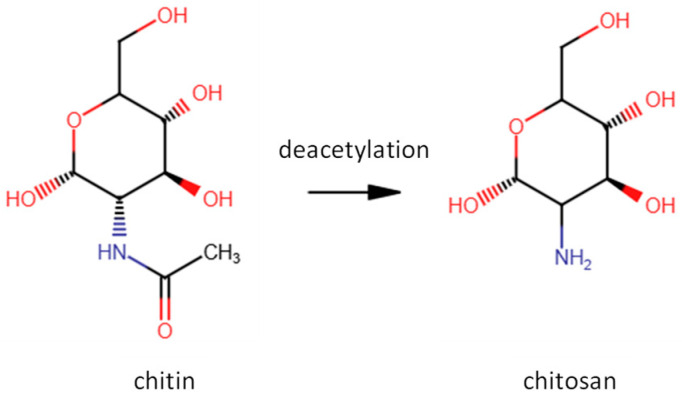
Deacetylation of chitin leading to the formation of chitosan through conversion of N-acetyl-D-glucosamine to D-glucosamine units.

Chitosan is characterized mainly by its molecular weight and degree of deacetylation (Figure 2). A higher degree of deacetylation increases the number of protonated amino groups, which strengthens electrostatic interactions and intermolecular cohesion. Similarly, higher molecular weight enhances mechanical strength through greater chain entanglement. However, these factors can also increase sensitivity to pH and moisture, potentially reducing stability under aqueous conditions [20,23,24].

In alginate–chitosan composites, the ratio between the two polymers plays a critical role. A balanced ratio favors effective charge pairing and the formation of a dense interconnected structure. In contrast, large deviations from this balance may weaken mechanical integrity due to excess electrostatic repulsion or insufficient interaction between chains [3,4].

### 2.2. Crosslinking Mechanisms and Processing Effects

The type and density of crosslinks play a central role in determining the mechanical performance of alginate–chitosan composites. Alginate typically undergoes ionic crosslinking in the presence of divalent cations such as calcium, leading to rapid gelation and the formation of reversible networks whose stiffness depends on crosslink density, ion concentration, and diffusion kinetics. In physiological or high-ionic-strength environments, these ionic crosslinks may weaken or be displaced by monovalent cations unless stabilized by additional interactions [3].

Chitosan can contribute to network stabilization through electrostatic complexation with alginate, chain entanglement, or, in some cases, covalent crosslinking introduced via chemical agents or surface modification strategies [22]. Processing approaches such as sequential gelation, layer-by-layer assembly, surface coating, or simultaneous complexation can produce markedly different morphologies, including homogeneous interpenetrating networks, layered or core–shell structures, and coacervate-type assemblies. These structural variations directly influence tensile strength, stiffness, elasticity, and resistance to deformation [25,26].

Processing conditions, particularly pH adjustment during synthesis, further modulate crosslink distribution and viscoelastic behavior by controlling the ionization state of functional groups and the extent of electrostatic interactions [27]. In addition to pH, other processing variables play a critical role in determining the microstructure and mechanical behavior of alginate–chitosan composites. The order of polymer addition can influence the spatial organization of polyelectrolyte complex (PEC) domains and the distribution of alginate-rich or chitosan-rich regions within the material. Different mixing pathways have been reported to produce distinct microstructures and particle sizes, which subsequently affect surface area, porosity, and colloidal stability [28,29]. Solvent composition and surfactant properties may also alter the arrangement of polymer chains at interfaces, thereby modifying pore architecture and mechanical integrity of the resulting materials [29]. Plasticizers may further modify intermolecular interactions and segmental mobility within the polymer matrix, thereby tuning film flexibility, stiffness, and mechanical strength depending on their type and concentration [30]. Furthermore, ionic strength plays an important role in regulating electrostatic complexation and ionic crosslinking. Increasing Ca^2+^ concentration promotes alginate crosslinking through egg-box domains and leads to denser networks with enhanced stiffness and elasticity, although excessive ionic strength may induce aggregation or brittle structures [28,31,32]. The presence of dissolved salts can additionally screen electrostatic interactions between alginate and chitosan chains, thereby modifying complexation behavior, phase organization, and the viscoelastic response of the resulting networks. Recent studies have shown that salt concentration can significantly influence the rheological and phase behavior of alginate–chitosan polyelectrolyte complexes, highlighting its role in tuning the mechanical performance of these materials [33]. In addition, the gelation or coating sequence can strongly affect particle size, encapsulation efficiency, and structural stability, as sequential Ca^2+^ crosslinking followed by chitosan complexation often produces more stable core–shell architectures [28,32]. These structural variations ultimately influence the mechanical performance of the resulting materials, including stiffness, elasticity, and structural stability [20,34].

### 2.3. Environmental Sensitivity

Alginate–chitosan composites are inherently sensitive to environmental conditions, especially moisture, pH, and ionic strength. Increased water uptake generally leads to swelling and a reduction in elastic modulus due to plasticization of the polymer network [21]. This effect can be partially mitigated through higher crosslink density, secondary coordination bonds, or the introduction of covalent crosslinks that restrict chain mobility.

pH plays a particularly important role in chitosan-containing systems. Lower pH values promote protonation of chitosan amino groups, strengthening electrostatic interactions with alginate and improving gel cohesion [20,34]. However, excessive swelling and mechanical weakening may still occur under prolonged exposure to aqueous or physiological conditions, highlighting the need for careful composite design and, in some cases, the use of reinforcing additives [21,35].

Reported swelling ratios for alginate–chitosan hydrogels typically range from 200% to 800%, although higher values exceeding 1000% have been reported depending on polymer composition, crosslink density, and environmental conditions [36,37,38]. Mechanical properties vary widely depending on formulation and structural design. Soft hydrogel and scaffold systems generally exhibit Young’s modulus values in the range of tens to hundreds of kilopascals [37,38], while compressive modulus values reported for reinforced hydrogel networks and composite scaffolds may reach the megapascal range depending on network architecture and reinforcement strategy [11,39,40]. By comparison, alginate–chitosan composite films and nanocomposites designed for structural or packaging applications often show tensile strength values in the MPa range, with reported values typically between 10 and 80 MPa depending on crosslinking strategy and filler incorporation [19,41,42]. Water uptake and equilibrium swelling behavior in these systems are closely related to network density and porosity. Increased crosslinking generally reduces mesh size and restricts water diffusion within the polymer matrix, thereby lowering swelling capacity, whereas more porous structures facilitate higher water absorption and swelling capacity [43,44,45,46]. These observations indicate that equilibrium swelling and water uptake are primarily governed by network density and pore architecture.

### 2.4. Application-Specific Mechanical Performance

Through careful adjustment of composition, crosslinking strategy, processing conditions, and environmental parameters, alginate–chitosan composites can display a broad range of mechanical behaviors. Compared with single-polymer systems, these composites often show improved flexibility, toughness, water resistance, and controlled degradation. Additional properties, such as antimicrobial activity, can also be introduced by incorporating nanoparticles or bioactive compounds.

As a result, alginate–chitosan composites have been widely explored for application-specific requirements, including wound dressings [12], tissue engineering scaffolds [10,24], drug delivery systems [16], food packaging films [25], and environmental remediation materials [35]. Their mechanical properties can be tuned either to resemble the behavior of biological tissues or to satisfy industrial performance requirements. This adaptability highlights the practical value of combining complementary polymer interactions within a single composite system.

## 3. Interpolymer Interactions and Network Formation in Alginate–Chitosan Composites

**Figure 3:**
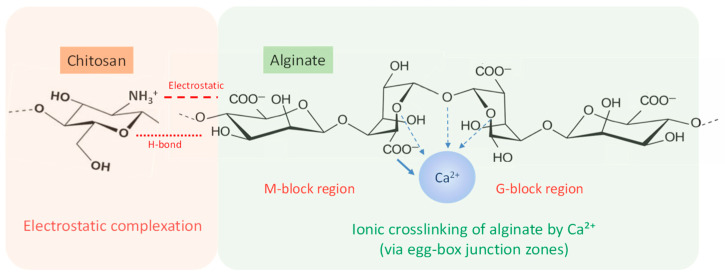
Interaction mechanisms governing network formation in alginate–chitosan composites. Electrostatic complexation occurs between alginate carboxylate groups (–COO^−^) and protonated amino groups of chitosan (–NH_3_^+^), while additional hydrogen bonding may occur between hydroxyl and amino groups of the two polymers. In parallel, Ca^2+^ ions coordinate with guluronic acid blocks of alginate to form ionic crosslinks through the characteristic egg-box junction zones.

Alginate–chitosan composites are well known for forming polyelectrolyte complexes through electrostatic attraction between the negatively charged carboxylate groups of alginate and the positively charged amino groups of chitosan [35], as illustrated in Figure 3. The mechanical behavior of these systems does not depend on a single type of interaction. Instead, it results from the combined effects of several interactions within the composite structure. These electrostatic associations occur spontaneously in aqueous environments and lead to the formation of interconnected polymer structures. The final organization depends strongly on factors such as charge balance, mixing procedure, pH, and ionic strength [2,4,17]. Experimental studies using techniques such as FTIR spectroscopy, zeta potential analysis, and microscopy have consistently confirmed the presence of ionic interactions between alginate and chitosan at their interface [4,13,47,48].

The formation of this interconnected network limits the mobility of polymer chains, which in turn reduces swelling and improves mechanical integrity compared to systems composed of a single polymer [4,35,49]. Different preparation approaches—including sequential gelation, surface coating, or simultaneous complexation—can produce a wide range of morphologies, such as homogeneous interpenetrating networks, layered or core–shell structures, and coacervate-type assemblies, each exhibiting distinct mechanical behavior [4,14,48].

At the molecular level, polyelectrolyte complex formation between alginate and chitosan is often initiated at the interface between oppositely charged chains and can propagate through diffusion-controlled rearrangement of polymer segments [4]. The resulting network architecture may involve competitive or cooperative interactions between alginate–calcium crosslinks and alginate–chitosan electrostatic associations, depending on the sequence of processing steps. In systems where calcium-induced gelation precedes chitosan addition, chitosan primarily acts as a reinforcing or surface-binding component, whereas simultaneous complexation can promote more homogeneous charge pairing throughout the bulk network [5].

Charge balance plays a key role in determining the final structure: compositions close to charge neutrality tend to form dense and mechanically stable networks, whereas excess charge from either polymer can result in heterogeneous architectures with reduced structural integrity [4,20]. Processing conditions further regulate crosslink distribution and network uniformity, thereby modulating overall mechanical response [4,35,49]. Together, these interaction-driven characteristics explain the highly tunable mechanical performance of alginate–chitosan composites and support their growing use in biomedical, environmental, and industrial applications [2,11,35].

## 4. Structural Parameters Governing Mechanical Properties

The mechanical behavior of alginate–chitosan composites depends mainly on their composition and processing conditions. Factors such as polymer ratio, charge balance, crosslinking characteristics, and molecular properties directly influence stiffness, strength, and resistance to deformation.

### 4.1. Polymer Ratio and Charge Balance

The alginate-to-chitosan ratio is a key parameter governing the extent and distribution of electrostatic interactions within composite systems. Near-stoichiometric charge conditions—where positive charges from chitosan closely balance the negative charges of alginate—favor the formation of compact and mechanically robust polyelectrolyte complexes. Under these conditions, strong electrostatic attraction effectively restricts chain mobility and enhances structural integrity [35,50,51]. Increasing chitosan content within an alginate matrix generally improves elastic modulus and tensile strength up to an optimal threshold. Beyond this point, excess polymer may lead to phase separation, swelling, or brittle behavior due to charge imbalance [9,15,51]. These findings underscore the importance of controlled compositional design to achieve materials that combine mechanical stability with sufficient deformability for practical applications [15,35,52,53].

### 4.2. Molecular Weight and Degree of Deacetylation

The molecular properties of chitosan, especially molecular weight (MW) and degree of deacetylation (DD), significantly affect the mechanical behavior of alginate–chitosan composites. Chitosan with higher MW generally promotes greater chain entanglement and stronger interactions between polymer chains, which often results in increased stiffness and tensile strength in films, hydrogels, and scaffold structures [6,8,54,55]. In a similar way, increasing DD raises the number of protonated amino groups, strengthening electrostatic interactions with polymers such as alginate or cellulose. Although stronger interactions can improve mechanical strength, they may also limit chain mobility and reduce flexibility when excessive [7,55,56,57].

The relationship between MW, DD, and mechanical properties is therefore not straightforward. Different applications require different molecular characteristics, as extremely high or low MW and DD values can negatively influence flexibility, porosity, or biological performance [58,59,60]. Beyond mechanical strength, MW and DD also affect hydrophilicity, degradation rate, crystallinity, and biocompatibility [1,7,61]. For this reason, a balanced selection of molecular parameters is essential. In many cases, composites prepared with high-MW chitosan and moderate to high DD provide a good compromise between strength and flexibility, particularly in tissue engineering scaffolds and packaging films [54,55,60]. For instance, scaffolds produced using medium-MW chitosan at moderate concentration have achieved compressive strength comparable to human cartilage while maintaining sufficient porosity for cell growth [60]. In some nanocomposite systems, DD appears to have a greater impact than MW on particle formation and charge density [7].

### 4.3. Crosslinking Strategies and Network Architecture

Crosslinking is a primary strategy for tuning the mechanical and functional properties of alginate–chitosan systems. The type, density, and spatial arrangement of junctions directly influence strength, elasticity, stability, and environmental responsiveness. Conventional approaches rely on either ionic or covalent bonding; however, the limitations of single-mode crosslinking—such as sensitivity to ionic conditions or restricted robustness—have encouraged the development of dual and sequential strategies. By integrating reversible ionic interactions with more permanent covalent bonds, these approaches enhance resilience and long-term stability [62,63,64,65]. Recent developments include the use of multi-arm crosslinkers that allow self-healing behavior to be adjusted [66], bio-based crosslinking agents such as tannic acid for multifunctional structures [62], and computational models that help predict structural organization and material behavior [67]. Together, these approaches influence how the composite is organized internally and, as a result, how it responds mechanically.

## 5. Reinforcement Strategies and Mechanical Enhancement

Beyond polymer composition and crosslinking, reinforcement has become an important strategy for further improving the mechanical properties of alginate–chitosan composites. The incorporation of micro- and nanoscale fillers can enhance stress transfer, restrict chain mobility, and modify the internal morphology of the material, leading to improved strength and stability.

### 5.1. Nanofillers and Load Transfer Mechanisms

Various nanofillers have been investigated as reinforcing components in alginate–chitosan systems. These include cellulose nanocrystals and nanofibrils [68,69], graphene oxide [70,71], carbon nanotubes [70], hydroxyapatite [72], silica nanoparticles [73], halloysite nanotubes [52], expanded perlite [74], and graphitic carbon nitride [19]. These fillers interact with the polymer matrix through hydrogen bonding, electrostatic attraction between alginate carboxylate groups and chitosan amino groups, and physical entanglement. As a result, they provide additional pathways for stress transfer within the composite.

In many studies, the addition of nanofillers has led to noticeable improvements in mechanical properties. Reported enhancements include increases in compressive modulus, tensile strength, Young’s modulus, elongation at break, and overall toughness, depending on filler type and concentration [11,19,21,68,70,72,74,75,76]. For instance, incorporating graphene oxide increased the breaking strength of chitosan–alginate films by about 70% [70]. The use of silicocarnotite fibers resulted in up to a 400% increase in compressive strength compared with unreinforced scaffolds [72]. Likewise, bacterial cellulose nanocrystals reduced pore size while increasing compressive strength from 0.189 MPa to 0.318 MPa [68].

### 5.2. Balancing Reinforcement and Network Functionality

While reinforcement is widely used to improve mechanical strength, it also influences several functional properties of polymer networks. Increased filler content often leads to reduced porosity and swelling, which may in turn decrease permeability and slow degradation rates, particularly in systems with dense networks or chitosan encapsulation [68,69,74,76]. In some cases, certain fillers also improve bioactivity. However, excessive filler addition may cause brittleness or reduce pore interconnectivity, limiting mass transport that is essential for applications such as tissue engineering and drug delivery.

Reinforcement strategies therefore need to be carefully adjusted to maintain a balance between mechanical strength and functional performance. In many systems, low to moderate filler loadings provide a reasonable compromise between improved strength and retained flexibility. Mechanical behavior can be further tuned through different crosslinking methods, including ionic, covalent, or coordination-based interactions. Layered or anisotropic designs may also be used to introduce directional strength without severely compromising porosity. In addition, incorporating multifunctional components, such as bioactive agents, can enhance mechanical properties while supporting favorable cellular responses [71].

Studies focused on application-oriented design and their reported mechanical performance metrics are summarized in Table 1, demonstrating how compositional design, crosslinking strategy, and reinforcement approaches collectively influence structure–performance outcomes across different application areas.

## 6. Structure–Mechanical Property Relationships

Alginate–chitosan composites are typically prepared through several fabrication strategies, including simple polyelectrolyte complexation, ionic gelation with multivalent cations such as Ca^2+^, layer-by-layer assembly, and sequential or hybrid crosslinking approaches. In many systems, alginate and chitosan solutions are mixed under controlled pH conditions to promote electrostatic complex formation, followed by additional ionic or covalent crosslinking to stabilize the network. Processing routes such as casting, freeze-drying, internal or external gelation, and multilayer assembly further influence pore architecture and structural homogeneity, which ultimately affect the mechanical performance of the resulting materials.

Studies reported in the literature indicate that the mechanical behavior of alginate–chitosan composites is governed primarily by structural organization rather than polymer chemistry alone. Rather than being governed by a single dominant interaction, macroscopic properties arise from the combined action of ionic, electrostatic, covalent, and filler-mediated constraints operating from the molecular to the microscale. Electrostatic attraction between oppositely charged chains establishes the primary load-bearing framework, while secondary interactions and crosslinking processes regulate stress redistribution and energy dissipation [22].

At the molecular level, alginate–chitosan systems are typically stabilized by polyelectrolyte complexation between carboxylate groups of alginate and protonated amino groups of chitosan. These ionic interactions form dynamic junction zones that can dissipate energy under deformation, contributing to the viscoelastic response of the network. When multivalent cations such as Ca^2+^ are introduced, alginate chains can additionally form “egg-box” structures in which guluronate residues coordinate the cations and create interchain junctions [22]. The coexistence of ionic alginate crosslinks and electrostatic alginate–chitosan complexes leads to hierarchical networks with multiple levels of connectivity [79]. Such multiscale organization plays a critical role in determining the balance between stiffness, elasticity, and toughness in composite systems. Mechanical response can therefore be interpreted in terms of the degree to which polymer chains are confined within the three-dimensional network.

**Figure 4:**
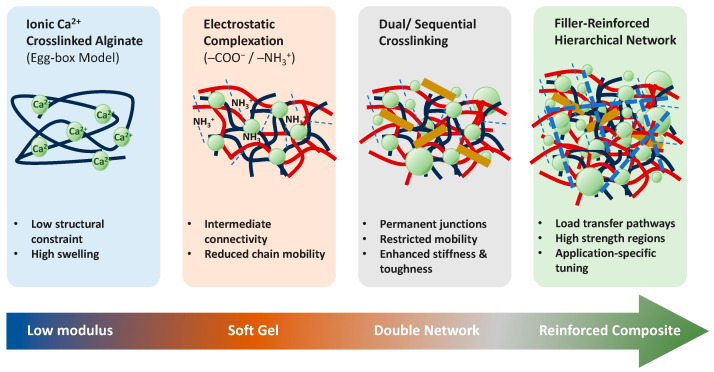
Hierarchical network evolution and progressive mechanical reinforcement in alginate–chitosan composites.

Highly interconnected structures typically exhibit increased stiffness and strength but reduced deformability. In contrast, loosely organized or heterogeneous architectures tend to display lower resistance to deformation while permitting greater flexibility and swelling [80]. These distinctions explain why systems with similar compositions may demonstrate markedly different mechanical characteristics depending on processing history and interaction hierarchy. This progression from soft ionic gels to more constrained composite architectures is schematically illustrated in Figure 4.

For example, reinforcement with bacterial cellulose nanofibers in alginate–chitosan scaffolds has been reported to increase compressive strength while preserving interconnected porosity, demonstrating how fiber–polymer interactions improve load transfer and stress distribution within the network [68]. Similar reinforcement strategies have also been reported in polysaccharide-based composites, where Schiff base crosslinking combined with graphene and nanocellulose reinforcement improved structural stability and mechanical integrity of chitosan-based foams for environmental applications [81]. In addition, graphene-based nanomaterials and other nanoscale fillers can significantly enhance tensile strength and elastic modulus in composite films through strong hydrogen bonding and improved stress distribution across the polymer matrix [71]. Reported mechanical properties of alginate–chitosan systems span a wide range depending on network architecture and reinforcement strategy. Soft hydrogel and scaffold systems typically exhibit compressive moduli ranging from tens to several hundred kilopascals, whereas reinforced composite scaffolds can reach values approaching the megapascal range [82]. In contrast, alginate–chitosan composite films designed for structural or packaging applications frequently display tensile strength values in the range of approximately 10–80 MPa depending on crosslinking density and filler incorporation [83]. Reported Young’s modulus values also vary widely depending on material format and hydration state: hydrated hydrogel systems generally exhibit moduli in the kilopascal to megapascal range, whereas dry multilayer or film-type structures may reach values in the gigapascal range due to dense polymer packing and reduced water content [10,26,84]. These studies collectively demonstrate that fillers influence alginate–chitosan composites not only by reinforcing the polymer matrix but also by modifying pore architecture, interfacial interactions, and stress distribution within the network, thereby significantly affecting the mechanical performance of the resulting materials.

Sequential or hybrid crosslinking strategies provide additional control by integrating interactions with different stability and reversibility. Ionic alginate domains introduce reversible junctions, whereas covalently stabilized chitosan segments act as persistent structural anchors that maintain network integrity. The combination of these mechanisms enables a balance between stiffness and toughness that is difficult to achieve through a single strategy [85,86]. Hybrid networks containing both ionic (Ca^2+^-mediated) and covalent crosslinks have been reported to improve structural stability while limiting excessive swelling, illustrating how dual crosslinking can enhance both modulus and mechanical resilience compared with purely ionically crosslinked systems [17]. Beyond increasing junction density, reinforcement strategies can further enhance mechanical response by creating additional load-transfer pathways without fundamentally altering chemical connectivity. The effectiveness of such reinforcement depends strongly on filler dispersion and interfacial compatibility. Rather than functioning merely as rigid inclusions, fillers redistribute mechanical loads and may enhance toughness or induce brittleness depending on concentration and polymer–filler interaction. In addition to filler reinforcement, network architecture itself can strongly influence mechanical response. Interpenetrating polymer networks (IPNs), multilayer assemblies, and core–shell structures have been shown to provide improved mechanical stability by combining networks with different deformation mechanisms, thereby enabling simultaneous control over stiffness, toughness, and permeability [82]. Optimizing performance is therefore not simply a matter of maximizing crosslink density or filler content. Increased structural confinement often reduces swelling and porosity, potentially compromising permeability or mass transport [11,41]. Mechanical optimization should instead focus on achieving a suitable balance between stiffness, strength, and functional requirements.

Collectively, the diverse outcomes reported in the literature can be understood as materials occupying different positions along a continuum of structural connectivity governed by composition, processing sequence, crosslinking approach, and reinforcement strategy. Recognizing these interdependencies supports a more rational pathway toward designing alginate–chitosan composites tailored to application-specific mechanical demands. This understanding also provides a critical foundation for tailoring alginate–chitosan composites to specific application requirements.

## 7. Application-Oriented Implications of Mechanical Design

The close link between structural design and mechanical properties is especially important when developing alginate–chitosan composites for specific applications. In tissue engineering, scaffolds need to be strong enough to maintain structural integrity while remaining flexible enough to resemble native tissues. At the same time, they must preserve sufficient porosity to allow cell infiltration and nutrient transport [10]. Carefully controlled crosslinking, combined with moderate reinforcement, can help achieve this balance.

In particular, tissue engineering scaffolds often require compressive moduli ranging from tens to several hundred kilopascals depending on the target tissue, while maintaining interconnected pore structures typically between approximately 70 and 200 μm to facilitate cell migration and tissue regeneration [71]. Reinforcement strategies involving nanocellulose fibers, hydroxyapatite, bioglass, or electrospun polymer nanofibers have been widely explored to increase compressive strength while preserving high porosity and biological functionality [11,76].

For wound dressings and drug delivery systems, mechanical integrity must be sufficient to maintain structural stability during handling and use, while still allowing swelling and controlled release [71]. In these situations, making the material too stiff can actually reduce its usefulness, which makes careful control of the internal structure essential. In contrast, applications such as food packaging and other sustainable materials often require higher tensile strength and greater resistance to deformation. Achieving these properties usually involves higher filler content or more compact structures [19,87]. These examples show that mechanical optimization should be guided by the specific needs of each application rather than by a single performance target. Wound dressing materials typically require flexible hydrogel or film structures capable of absorbing wound exudate while maintaining mechanical integrity. Swelling ratios are often maintained at moderate levels to allow fluid uptake without compromising structural stability [88]. Ionic crosslinking using Ca^2+^ or Zn^2+^ ions and adjustment of the alginate–chitosan ratio are commonly employed to regulate swelling behavior while maintaining adequate tensile strength and elasticity [30]. In addition, the incorporation of chitosan nanoparticles, antibacterial nanofillers, or multilayer film architectures can further enhance both mechanical robustness and antimicrobial performance in wound healing applications [89,90].

In drug delivery systems, mechanical stability plays an important role in maintaining structural integrity during administration and throughout the release process. Hydrogels or microcapsules that are too mechanically weak may degrade prematurely, while overly dense networks may hinder the diffusion of therapeutic molecules. To address this challenge, core–shell architectures and microparticle-in-hydrogel systems have been developed, where Ca^2+^-crosslinked alginate microspheres are embedded within chitosan-based matrices [91]. These hierarchical structures provide improved compressive stability while simultaneously creating controlled diffusion pathways for sustained drug release. In some injectable hydrogel systems, compressive moduli of approximately 50–100 kPa have been reported, enabling adequate structural stability while preserving sufficient network permeability for therapeutic delivery [71].

In contrast, applications such as food packaging and other sustainable materials often require higher tensile strength and greater resistance to deformation. Achieving these properties usually involves higher filler content or more compact structures [19]. For biodegradable packaging films, tensile strength values can vary widely depending on crosslink density and filler reinforcement. Alginate–chitosan films typically exhibit tensile strengths in the range of several megapascals, while incorporation of nanofillers such as graphene oxide, carbon nanotubes, or graphitic carbon nitride can significantly increase tensile strength, in some cases exceeding tens of megapascals [18,19]. These nanofillers improve mechanical resistance by strengthening interfacial interactions and reducing polymer chain mobility, while also improving barrier properties against water vapor and oxygen. Multilayer film architectures and strong polyelectrolyte complexation between alginate and chitosan further contribute to improved mechanical stability and reduced water uptake in packaging materials.

These examples show that mechanical optimization should be guided by the specific needs of each application rather than by a single performance target. Successful design of alginate–chitosan composites requires matching network architecture, crosslinking strategy, filler reinforcement, and swelling behavior to the mechanical and environmental conditions of the intended application. By tailoring these structural parameters, alginate–chitosan systems can be engineered to provide soft, elastic networks for biomedical hydrogels, mechanically stable scaffolds for tissue regeneration, controlled-release matrices for drug delivery, or strong and low-permeability films for sustainable packaging technologies.

## 8. Current Challenges and Future Perspectives

Although considerable progress has been made, several challenges still limit the broader use of alginate–chitosan composites in advanced material applications. Variations in polymer source, molecular weight distribution, and degree of deacetylation often lead to inconsistent mechanical outcomes. For instance, variations in alginate M/G ratio or chitosan degree of deacetylation can significantly influence gelation behavior, viscosity, crosslinking density, and ultimately the stiffness and swelling characteristics of the resulting networks [92]. Such variability often results in batch-to-batch differences in mechanical strength, degradation rate, and bioactivity, which complicates reproducibility and large-scale implementation [2]. In addition, the lack of standardized mechanical testing protocols makes direct comparison between different studies difficult.

Another important limitation concerns the intrinsic mechanical weakness and swelling sensitivity of ionically crosslinked alginate–chitosan networks. Conventional Ca^2+^-mediated alginate crosslinking forms reversible junctions that can be destabilized by monovalent salts or chelating agents, often leading to excessive swelling, structural relaxation, and mechanical degradation under physiological or environmental conditions [76]. In highly porous scaffold systems, this swelling–softening effect can compromise load-bearing capacity and long-term mechanical stability. Similarly, in packaging or environmental systems, repeated swelling–deswelling cycles may cause structural fatigue and gradual loss of functional performance [68].

Crosslinking strategies therefore remain a central research challenge. While ionic crosslinking provides rapid gel formation and mild processing conditions, the resulting networks are often mechanically weak and sensitive to ionic environments. Conversely, covalent crosslinking can significantly enhance stiffness and structural stability but may introduce toxicity concerns when conventional agents such as glutaraldehyde are used [93]. Developing crosslinking approaches that combine mechanical robustness, cytocompatibility, and processability—such as hybrid ionic–covalent systems, natural crosslinkers, or dynamic covalent interactions—remains an important direction for future work.

Future research should focus on achieving better control over network formation through well-defined processing conditions and clearly established structure–property relationships. In particular, systematic studies that link polymer composition, crosslinking density, filler reinforcement, and network architecture to quantitative mechanical metrics such as elastic modulus, toughness, and swelling ratio are still limited. Establishing predictive structure–process–property relationships would greatly facilitate the rational design of alginate–chitosan composites tailored to specific mechanical and functional requirements. In this context, multiscale characterization approaches that integrate molecular-level information with microstructural and macroscopic mechanical analyses are particularly valuable, as they provide a more complete understanding of how processing and structure influence performance.

Recent research also highlights the importance of advanced network architectures. Double-network hydrogels, semi-interpenetrating polymer networks (semi-IPNs), and hierarchical porous structures have shown promising improvements in mechanical strength, toughness, and functional stability [40,69]. These architectures allow the decoupling of stiffness, toughness, and swelling behavior by integrating networks with different deformation mechanisms, thereby enabling materials that are simultaneously strong and flexible. Incorporation of nanoscale fillers such as nanocellulose, graphene derivatives, or mineral particles can further enhance mechanical reinforcement through improved load transfer and interfacial interactions [69].

Moreover, for applications that require long-term mechanical stability, the development of scalable and reproducible manufacturing strategies will be essential to enable reliable industrial implementation. Many high-performance alginate–chitosan composites are currently fabricated using laboratory-scale techniques such as freeze-drying, layer-by-layer assembly, or cryostructuring, which can be energy-intensive and difficult to scale. Future efforts should therefore emphasize continuous and industrially compatible processing methods, including extrusion, film casting, coating technologies, and advanced additive manufacturing approaches such as 3D printing [94,95]. Improving rheological control and crosslinking kinetics during processing will also be crucial for maintaining uniform structure and mechanical performance at larger scales. Future research trends indicate that alginate–chitosan composites are increasingly being designed as multifunctional and adaptive materials. Hybrid crosslinking strategies that combine ionic, covalent, and supramolecular interactions are being explored to achieve tunable mechanical properties, self-healing behavior, and environmental responsiveness. In addition, sustainable processing routes and greener crosslinking chemistries are receiving growing attention, particularly for applications in food packaging, environmental remediation, and agricultural delivery systems. Progress in this field will therefore depend on integrating molecular design, multiscale structural characterization, and scalable manufacturing approaches to enable the reliable production of alginate–chitosan composites with predictable mechanical performance and application-specific functionality.

## 9. Design Considerations for Tailoring Mechanical Performance

The studies reviewed here indicate that achieving targeted mechanical properties requires a design-oriented approach in which composition, interaction type, and processing sequence are selected according to the intended mechanical regime rather than treated as independent formulation variables. Because these materials derive their behavior from hierarchical interactions, minor variations in preparation conditions can produce substantial differences in stiffness, toughness, and environmental stability.

Across alginate–chitosan systems, mechanical performance is primarily governed by several coupled design parameters, including polymer ratio and charge balance, crosslinking mode and density, incorporation of reinforcing phases, and processing sequence. These factors collectively determine network density, pore architecture, swelling behavior, and long-term mechanical stability.

From a practical standpoint, adjusting the **alginate-to-chitosan ratio** toward charge balance promotes effective complex formation and structural densification. Deviations from this balance may be intentionally employed to obtain softer or more swellable materials, although often at the expense of strength. Polymer composition thus serves as a primary tool for positioning the material within a desired connectivity range. Near-stoichiometric charge conditions generally maximize electrostatic complexation and mechanical performance, whereas significant charge imbalance can weaken the network due to electrostatic repulsion or incomplete complex formation. Slightly alginate-rich compositions tend to produce more flexible and swellable materials, while chitosan-rich systems often exhibit higher thermal and mechanical stability due to increased network densification.

The chosen **crosslinking strategy** then determines the permanence of this structure. Ionic gelation alone is suitable for applications requiring reversibility and high deformability, whereas sequential or dual crosslinking enhances resistance to deformation and long-term stability. In practice, ionic alginate–chitosan complexes may be further strengthened through secondary crosslinking mechanisms such as Ca^2+^ ionotropic gelation, covalent crosslinking (e.g., Schiff-base reactions or genipin-mediated bonds), or hybrid systems combining ionic and covalent interactions. Such hybrid networks typically exhibit reduced swelling, improved dimensional stability, and higher modulus compared with purely ionically crosslinked systems [22,91].

Processing sequence plays a decisive role, as it dictates whether interactions cooperate or compete within the final architecture. For example, homogeneous complexation strategies—such as gradual pH adjustment or internal gelation approaches—can produce more uniform networks and improved mechanical performance compared with rapid external gelation that often results in heterogeneous structures or weak surface layers [68]. Similarly, multistep fabrication approaches including layer-by-layer assembly or post-crosslinking treatments enable better control over film thickness, interfacial interactions, and overall mechanical strength [30].

**Reinforcement strategies** should be implemented with careful consideration of both strength and functionality. Moderate filler incorporation can improve load transfer and limit excessive swelling, whereas excessive loading may suppress chain mobility and promote brittleness. Optimal performance is typically achieved when reinforcing elements complement the existing polymer assembly rather than dominate it. A wide range of reinforcing phases—including bacterial cellulose nanocrystals, nanocellulose fibers, graphene-based nanomaterials, carbon nanotubes, and inorganic fillers such as hydroxyapatite or bioglass—have been shown to substantially increase tensile strength, compressive modulus, and toughness while simultaneously reducing swelling and improving environmental stability [76,96]. However, these benefits strongly depend on filler dispersion and interfacial compatibility within the polymer network.

**Environmental sensitivity** should not only be seen as a limitation but also as a factor that can be deliberately adjusted during material design. By modifying crosslink density and the types of interactions within the structure, it is possible to maintain mechanical stability under specific pH, ionic strength, or moisture conditions. For instance, denser networks formed through higher crosslink density or rigid fillers typically exhibit reduced pore size, lower water uptake, and improved dimensional stability, although this may restrict diffusion or permeability depending on the intended application [10,70].

As a result, alginate–chitosan composites can be developed as soft viscoelastic gels, durable films, or load-bearing scaffolds, depending on the intended application. Design strategies must therefore align mechanical targets with structural parameters: highly swellable and deformable ionic networks are often preferred for injectable hydrogels or controlled drug delivery systems, whereas covalently or hybrid-crosslinked networks reinforced with nanofillers are more suitable for mechanically demanding applications such as tissue engineering scaffolds, packaging films, or structural biopolymer composites.

## 10. Conclusions

Alginate–chitosan polymer composites constitute a versatile class of bio-based materials whose mechanical characteristics can be deliberately adjusted through compositional control and interaction design. The literature demonstrates that polymer ratio, charge balance, crosslinking strategy, and reinforcement collectively shape the internal organization of these systems and thereby influence stiffness, strength, deformability, and long-term stability. Mechanical behavior in these composites depends not only on the type of polymers used but also on how their interactions are formed and distributed within the structure. Understanding this relationship makes it easier to adjust mechanical properties in a controlled way and to adapt the materials for different applications, including biomedical devices and sustainable packaging.

Despite these advances, several challenges remain that limit the broader translation of alginate–chitosan composites into practical technologies. Future research should focus on improving mechanical robustness without sacrificing porosity or mass transport, particularly in applications such as tissue engineering scaffolds where structural integrity and biological performance must be balanced. Emerging strategies such as hierarchical reinforcement, multifunctional nanofillers, and double-network or hybrid architectures offer promising routes to decouple stiffness, toughness, and swelling behavior.

Another important direction involves the development of greener and more versatile crosslinking strategies. While ionic gelation and conventional chemical crosslinkers remain widely used, recent work highlights the potential of hybrid crosslinking systems and self-crosslinking chemistries that combine ionic, covalent, and supramolecular interactions to achieve improved stability and responsiveness. In parallel, advances in scalable fabrication techniques—including rheology-guided 3D printing, extrusion, and coating technologies—are expected to facilitate reproducible and application-oriented manufacturing of alginate–chitosan materials.

These insights demonstrate that the mechanical behavior of alginate–chitosan composites can be rationally tuned by coordinating composition, crosslinking strategy, reinforcement, and processing conditions, thereby enabling the development of materials tailored for diverse applications ranging from soft hydrogels to mechanically robust scaffolds and films.

## Figures and Tables

**Table 1:** Reported Structure–Performance Relationships in Alginate–Chitosan Composite Systems.

**Tissue Engineering and Regenerative Materials**			
**Study Focus**	**Evaluated Properties**	**Key Findings**	**Reference**
Bone tissue scaffolds **(Scaffold fabrication)**	Porosity, mechanical properties, biodegradation	Bacterial cellulose improved pore architecture, swelling control, and cytocompatibility	[68]
Bioactive biomimetic scaffolds **(Crosslinked scaffold)**	Mechanical integrity, biomimetic structure	Crosslinking increased strength (~30 MPa) and enhanced cell proliferation.	[10]
Composite scaffolds with bacterial cellulose **(Reinforced scaffold)**	Mechanical properties, swelling, cytocompatibility	Reinforcement enhanced scaffold strength and protein adsorption.	[69]
Injectable composite systems **(Hydrogel)**	Biocompatibility, structural stability	High injectability and >95% cell viability with strong tissue adhesion	[71]
Aminated composite scaffolds **(Functionalized scaffold)**	Mechanical strength, cytocompatibility	Amination improved compressive strength and cell attachment	[52]
Bioglass-reinforced composites **(Hybrid scaffold)**	Mechanical performance, degradation	Improved mineralization and mechanical strength for bone regeneration.	[77]
Alginate–chitosan 3D supports **(Scaffold)**	Cell adhesion, physicochemical stability	Demonstrated excellent biocompatibility for cell culture systems.	[76]
**Drug Delivery and Biomedical Functional Systems**			
**Study Focus**	**Evaluated Properties**	**Key Findings**	**Reference**
Bilayer wound-dressing systems (Film/membrane)	Drug release, antimicrobial activity	Sustained drug release (60–70% over 96 h) with antimicrobial function.	[14]
pH-responsive composite networks **(Hydrogel)**	Viscoelasticity, mechanical strength	Gelation and encapsulation behavior tunable via pH and composition.	[20]
**Sustainable Packaging and Barrier Materials**			
**Study Focus**	**Evaluated Properties**	**Key Findings**	**Reference**
Flexible packaging materials **(Film)**	Mechanical and thermal properties	Increased chitosan improved flexibility and thermal resistance.	[4]
Nanocellulose-reinforced systems **(Nanocomposite film)**	Barrier and mechanical behavior	Barrier performance improved by ~45% against oxygen and moisture	[18]
Multifunctional packaging composites **(Nanocomposite film)**	Mechanical strength, permeability	Tensile strength increased (~19%) with strong UV shielding.	[19]
Composite aerogels for active packaging **(Aerogel)**	Mechanical and antimicrobial properties	Enhanced antibacterial activity and thermal stability observed	[35]
**Advanced Composite Reinforcement Strategies**			
**Study Focus**	**Evaluated Properties**	**Key Findings**	**Reference**
Polyurethane-based biocomposites **(Polymer composite)**	Structural and thermal properties	Small alginate additions improved thermal stability (~20 °C).	[78]
Alginate gels crosslinked with chitosan oligomers **(Ionically crosslinked gel)**	Gel strength, network structure	Poly-MG systems showed significantly stronger gel networks	[22]
Wet-spun composite fibers **(Fiber fabrication)**	Mechanical strength, rheology	High tensile strength and antibacterial properties achieved	[49]

## Data Availability

No new data were created or analyzed in this study. Data sharing is not applicable to this article.
